# Parent–offspring conflict and the genetic trade-offs shaping parental investment

**DOI:** 10.1038/ncomms7850

**Published:** 2015-04-16

**Authors:** Mathias Kölliker, Stefan Boos, Janine W.Y. Wong, Lilian Röllin, Dimitri Stucki, Shirley Raveh, Min Wu, Joël Meunier

**Affiliations:** 1 Department of Environmental Sciences, Zoology and Evolution, University of Basel, Vesalgasse 1, 4051 Basel, Switzerland; 2 Center of Excellence in Biological Interactions, Department of Biosciences, University of Helsinki, Viikinkaari 1, 00014 Helsinki, Finland; 3 Department of Evolutionary Biology, Institute of Zoology, Johannes Gutenberg University of Mainz, 55128 Mainz, Germany

## Abstract

The genetic conflict between parents and their offspring is a cornerstone of kin selection theory and the gene-centred view of evolution, but whether it actually occurs in natural systems remains an open question. Conflict operates only if parenting is driven by genetic trade-offs between offspring performance and the parent's ability to raise additional offspring, and its expression critically depends on the shape of these trade-offs. Here we investigate the occurrence and nature of genetic conflict in an insect with maternal care, the earwig *Forficula auricularia*. Specifically, we test for a direct response to experimental selection on female future reproduction and correlated responses in current offspring survival, developmental rate and growth. The results demonstrate genetic trade-offs that differ in shape before and after hatching. Our study not only provides direct evidence for parent–offspring conflict but also highlights that conflict is not inevitable and critically depends on the genetic trade-offs shaping parental investment.

## Abstract (web summary)

Sexual reproduction introduces genetic conflict between family members, but direct empirical evidence is lacking. Here, the authors show, in an insect with maternal care, that genetic trade-offs that differ in shape across offspring stages affect the scope for parent–offspring conflict.

## Graphical abstract

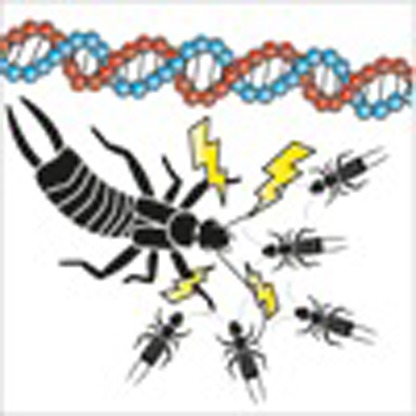

Parenting takes time, resources and energy, and ultimately reduces the parent's ability to produce additional offspring. It only pays off evolutionarily because it enhances the fitness of offspring to which the parent is genetically related^1^. But parenting is not necessarily harmonious altruism. Sexual reproduction is thought to introduce genetic conflict between family members. Each offspring should demand more investment than parents are selected to provide because it is more related to itself than to any of its siblings, whereas parents are equally related to all of their offspring^2^. Although the premise of parent–offspring conflict was conceptually quickly confirmed and accepted after Trivers' original formulation in 1974^3^,^4^,^5^, almost two decades later the lack of empirical tests was striking and the topic considered a ‘case of arrested development'^6^. Godfray^7^ identified the lack of testable predictions of the theory as the main problem and proposed a major shift in the research programme away from the conflict as such (that is, the ‘conflict battleground'^7^) to how parents and offspring should behave to resolve conflict^7^,^8^,^9^. This approach triggered a great amount of experimental research on behavioural parent–offspring interactions that provided evidence broadly consistent with conflict (reviewed in refs 5, 10, 11, 12, 13, 14). However, the downside of this approach was that it sidestepped the fundamental question whether genetic parent–offspring conflict actually occurs and, thus, whether its assumed prominent role as driver of parenting and family life is justified.

There are three main predictions that empirical tests of a Triversian parent–offspring conflict battleground have to address. First, the conflict is over parental investment (PI) and not over parenting behaviour. Thus, it is essential to quantify PI according to its ultimate definition, that is, to measure any investment by a parent that enhances offspring fitness at the expense of the parent's expectation for additional offspring (Fig. 1)^1^,^2^,^4^,^15^. Second, the conflict is among genes, not traits or behaviours, and therefore only occurs if PI is shaped by genetic rather than phenotypic trade-offs between parents and offspring. Hence, empirical tests should demonstrate that genotypes with enhanced performance as offspring exhibit reduced ability to raise many offspring as parents (due to higher PI), and *vice versa* for genotypes with reduced performance as offspring^16^,^17^. Finally, while genetic trade-offs provide evidence for antagonistic parent–offspring co-evolution, they *per se* are not sufficient evidence for parent–offspring conflict over the amount of PI. This conflict occurs when PI fitness optima differ for parent and offspring^2^,^7^,^14^, a condition requiring sexual reproduction and depending on the shape of the genetic trade-offs. It is only occasionally reached when offspring fitness gains show constant or accelerating returns, but always met under diminishing returns, that is, when offspring stand to gain less from an additional unit of investment when they are already in good than when they are in poor condition (Fig. 1)^2^,^4^,^5^,^18^. Hence, experimental tests should investigate the presence and shape of the genetic trade-offs, with evidence for conflict being most compelling under diminishing returns.

Theoretically, PI contains on the one hand the trade-off between investment in current offspring and the parent's expectation of future offspring (potentially leading to between-clutch conflict), and on the other hand the reallocation of investment among offspring within clutches (potentially leading to within-clutch conflict)^5^,^19^,^20^,^21^. In this study, we focused on the former and tested the three above predictions using a large scale and replicated selection experiment in an insect with extended maternal care, the earwig *Forficula auricularia*. The genetic trade-offs shaping PI were investigated by exerting selection on the mothers and quantifying the correlated responses in offspring. *F. auricularia* is an ideal system for this study: the species reproduces sexually (a prerequisite for parent–offspring conflict^2^), females care for eggs and hatched nymphs, and they produce up to two clutches in their lifetime^22^,^23^,^24^. From the viewpoint of earwig females, first-clutch offspring are current offspring, the relative size of the second clutch is an estimate of the female's expectation for future offspring, and the relationship between the size of the second clutch and the performance of first-clutch offspring quantifies the trade-offs shaping PI. Finally, multiple paternity is common in earwigs^25^, leading to variation in genetic relatedness within and between first and second clutches that can further mediate scope for conflict.

We selected females with low expectation of future offspring (that is, **S**mall relative size of (or no) second clutch; **S**-lines), high expectation of future offspring (that is, **L**arge relative size of second clutch; **L**-lines) and intermediate expectation of future offspring (that is, **C**ontrol; **C**-lines) in ten independent experimental populations over the course of six generations. The experiment included a total of 2,720 females with their offspring (287,636 eggs and 214,815 nymphs of first and second clutches). We predicted a correlated response to selection in offspring performance that was antagonistic to the direct response in females, with increased performance in S-lines and decreased performance in L-lines. Offspring performance was followed by covering the periods of maternal care before and after hatching and including measures of developmental rate, growth and survival. Finally, we explored the shape of the genetic trade-offs emerging between selection lines in the last generation. Overall, our results demonstrate (1) the occurrence of genetic trade-offs between the mother's expectation of future offspring and several offspring performance traits expressed before and after hatching; and (2) diminishing returns for offspring performance before hatching, but constant returns after hatching when mothers and offspring interact. Our study provides clear evidence for a parent–offspring conflict battleground during the egg stage, and highlights that its occurrence and nature critically depends on the genetic trade-offs shaping PI.

## Results

### Direct response to selection in mothers

S-line females evolved towards a lower relative second-clutch size as compared with L-line females (Fig. 2a), as expected. Per generation, the S- and L-lines diverged by 0.106 s.d. units (Fig. 2b) resulting in a mean difference of 0.637 s.d. in generation six (Fig. 2a). This response was due to significant changes in the size of the second clutch, while the size of the first clutch did not change significantly (Table 1). Furthermore, S-line females gained significantly less mass within 14 days after hatching of their first clutch (Table 1), a morphological proxy predicting second-clutch production^24^. These findings together confirm that S-line females evolved lower expectation for future offspring production than L-line females.

### Correlated responses to selection in offspring

Four performance traits of first-clutch offspring showed the antagonistic correlated responses to selection expected under a genetic trade-off. During the egg stage, hatching success and the rate of embryonic development increased in the S-line compared with the L-line (Fig. 2c,d; Table 1). The effect on hatching success was partly mediated by filial cannibalism, as L-line females showed an increasing tendency for egg cannibalism compared with C- or S-line females (Table 1). After hatching, early nymph survival and their relative mass gain until day 14 showed the expected correlated responses, increasing in the S-lines relative to L-lines (Fig. 2e,i). The correlated responses in early and late nymph developmental rate were not significant (Fig. 2f,g) and nymph body mass at hatching decreased, rather than increased, in S-lines (Fig. 2h).

### Shape of the genetic trade-offs

The shape of the trade-off curves was inferred from the relationships between the population means for the size of second clutches and the offspring performance traits across the three selection treatments (Fig. 3). Only the data from the last generation were used because the likelihood to detect diminishing returns, if present, is highest when mean trait values have diverged most. Qualitative evidence for a concave curved genetic trade-off and, thus, for diminishing returns and conflict was found for the egg stage in relation to hatching success and embryonic developmental rate (Fig. 3a,b). In contrast, the trade-offs after hatching with mass gain and nymph survival were approximately linear and indicated constant rather than diminishing returns (Fig. 3c,d). The slope with nymph mass gain was less steep than −1 (slope=−0.63), but steeper than −1 (while also less clearly linear) with regard to nymph survival (slope=−1.37).

## Discussion

Behaviours in families are generally thought to be the outcome of a genetic conflict over parental investment. This conflict is a cornerstone of kin selection theory and the gene-centred view of evolution^2^,^7^,^26^. However, an empirical demonstration of the conflict battleground^7^ has remained an unsolved difficulty to this day, partly due to intrinsic limitation of behavioural or phenotypic studies to demonstrate genetic conflict^6^,^12^,^14^, and partly due to experimental difficulties of quantifying PI^27^ and demonstrating different fitness optima for parents and offspring^7^,^14^.

In this study, we addressed these open questions using a selection experiment in the earwig *F. auricularia* and show empirical evidence for genetic conflict between parent and offspring over PI, at least during the egg stage. More specifically, we show that experimentally selecting on the females' expectation for future offspring (that is, the relative size of their second clutch) resulted in a direct response in terms of second-clutch size and correlated antagonistic responses to selection in offspring performance traits. These results demonstrate genetic trade-offs shaping PI, which is an essential (albeit not sufficient; see introduction) precondition for conflict to occur. The direct and correlated responses to selection were consistent among replicate lines with small and nonsignificant variation between population pairs due to drift. Furthermore, different fitness optima for earwig mothers and offspring were inferred by examining the shape of the genetic trade-offs in the last generation. They showed diminishing returns during the egg stage revealing scope for parent–offspring conflict over hatching success and egg developmental rate. After hatching, the trade-offs were linear implying constant returns and a probably minor role for conflict over nymph survival and growth (see below).

The correlated responses to selection in offspring were in the direction predicted by genetic trade-offs with regard to four offspring performance traits. As compared with L-line offspring, S-line offspring evolved towards enhanced hatching success, faster egg development, higher nymph survival and mass gain. The trade-off with hatching success was partly due to L-line females evolving a higher tendency to cannibalize their eggs, which fits the expectation that females with higher expectation for future reproduction should prioritize somatic maintenance (that is, food intake by egg recycling) over parenting and current offspring survival^1^,^15^. The responses in egg developmental rate may be due to changes in maternally transferred hormones or resources in the eggs, which are common maternal effect mechanisms across taxa^28^,^29^,^30^, or in maternal egg care behaviour^31^. The correlated responses of nymph survival and growth indicate enhanced post-hatching maternal care in S-line females, for example, through food provisioning^23^,^32^ and/or maternal modulation of siblicide among nymphs. In earwigs, nymph mortality is partly due to siblicide^33^ and, thus, the enhanced survival of nymphs in the S-line could also indicate a reduced siblicidal tendency of S-line nymphs. Compared with these four traits, the correlated response to selection in nymph body mass at hatching is less straightforward to interpret leaving room for two alternative interpretations. It could either reflect more maternal care during the egg stage by S-line females because attended eggs are known to develop into lighter hatchlings than orphaned eggs^31^, possibly due to the selective survival of heavier hatchlings under low levels of egg care. In this case, the observed response would be according to the predictions of a trade-off. Alternatively, because hatchlings from smaller eggs tend to be lighter^34^, S-line females may produce smaller eggs, which would be opposite to prediction. Given the straightforward interpretation of the first four offspring performance traits as components of the genetic trade-offs shaping PI, we focused on these in our examination for diminishing returns and scope for conflict.

The shape of the genetic trade-offs was inferred by comparing the evolutionarily diverged offspring performance traits and relative size of the females' second clutches between the three selection treatments. The curved genetic trade-offs during the egg stage indicate diminishing returns providing evidence for conflict over hatching success and egg developmental rate. Specifically, the increase in hatching success/developmental rate per unit decrease in the size of the female's second clutch was less between the C- and S-lines (high offspring performance) than between the C- and L-lines (low offspring performance). At first view, conflict during the egg stage may be thought to have little evolutionary consequence because the eggs are developmentally constrained in their ability to influence PI, and part of the conflict was due to female filial cannibalism that eggs cannot prevent. However, embryos are known to respond developmentally to other, more subtle forms of maternal influences (for example, maternal hormones in the eggs), and conflict can operate on these mechanisms^29^,^30^. The potential occurrence, scope and function of such maternal effect mechanisms remain to be investigated in *F. auricularia*.

**Figure 1:**
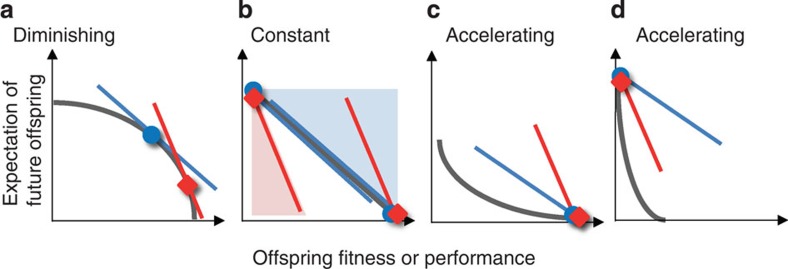
Theoretical plots depicting how the shape of genetic trade-offs affect the parent–offspring conflict battleground. (**a**) Curved trade-off with diminishing returns (grey line). The intersection of the fitness isoclines (tangent lines) to this curve are optima and their slope is steeper for the offspring (red line) than for the parent (blue line) because each offspring is at least twice as related to itself than to its sibling, whereas the parent is equally related to all its offspring (slope for parent=−1; slope for offspring=−2 in case of full siblings^4^). The parent and offspring optima (blue circle and red diamond, respectively) differ and, thus, there is parent–offspring conflict over the amount of PI in current offspring (modified from ref. 4). (**b**) Linear trade-off with constant returns. When the trade-off lines have slopes that lay in the blue area, parent and offspring agree that the parent should not produce future offspring. Conversely when the trade-off lines have slopes that lay in the red area, parent and offspring agree that the parent should terminate PI and produce additional offspring. When the trade-off lines have slopes equivalent to the fitness isoclines, no optima occur and all combinations of parent and offspring values are equivalent. Only in the white area there is conflict; not over the quantitative partitioning of PI among offspring, but over whether or not future offspring should be produced. (**c**,**d**) Curved trade-off with accelerating returns. (**c**) When current offspring stand to gain substantially from PI, the parent should invest all its resources in current offspring, produce no future clutch and there is no conflict. (**d**) When current offspring do not gain much from further PI, the parent should terminate its investment, produce a second clutch and there is no conflict. Conflict can only occur for trade-off curves intermediate to (**c**) and (**d**); not over the quantitative partitioning of PI among offspring, but over whether or not future offspring should be produced.

**Figure 2:**
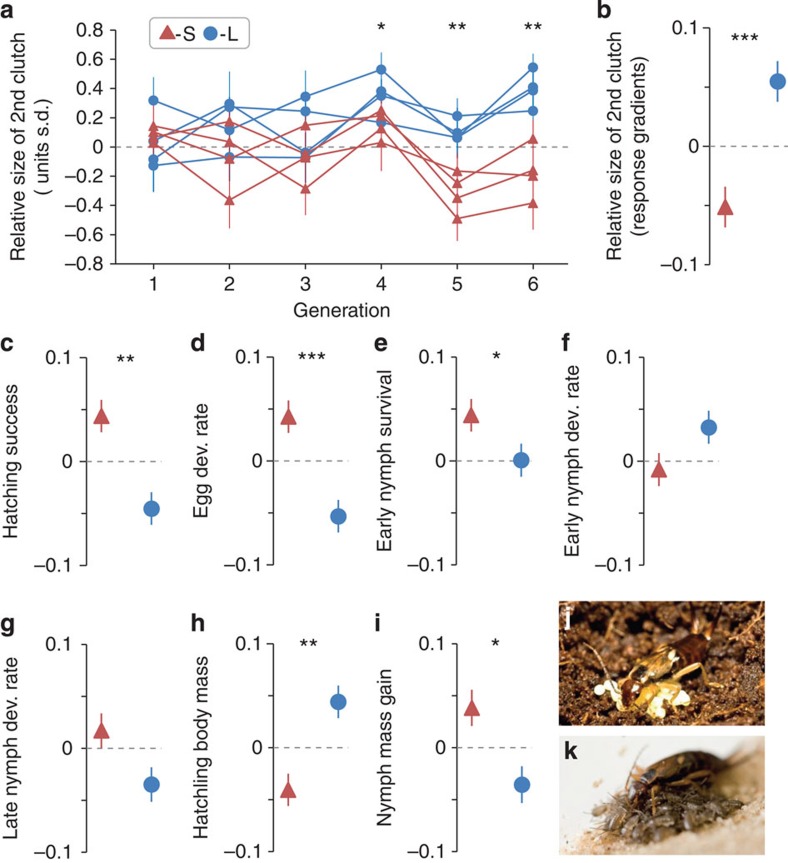
Direct and correlated responses to selection. *N*=4 S-lines (red symbols and lines), *N*=2 C-lines and *N*=4 L-lines (blue symbols and lines) throughout. Direct response to selection as (**a**) time course of the mean (±s.e.m.) trait values per replicate selection line (population pair), computed as deviation from the mean of the two control (C) lines and (**b**) as linear response gradients (estimated using linear mixed models (LMMs); see ‘Statistical analysis' in Methods section and Table 1; *n*=2,289 females with offspring). The correlated responses to selection in first-clutch offspring are displayed as linear response gradients: (**c**) proportion of hatched eggs (*n*=2,628); (**d**) egg developmental (dev.) rate between oviposition and hatching (*n*=2,519); (**e**) proportion of nymphs surviving from hatching until day 14 (*n*=2,474); (**f**) early nymph developmental rate from hatching to molt to second instar (*n*=2,438); (**g**) late nymph developmental rate from second instar to adult emergence (*n*=2,228); (**h**) mean nymph body mass 1 day after hatching (*n*=2,507); and (**i**) proportional nymph mass gain from hatching until day 14 (*n*=1,415). The scales on the y-axes are in units of s.d. *P<0.05, **P<0.01, ***P<0.001; LMM. (**j**) Picture of an earwig female tending her eggs, and (**k**) of a female tending her nymphs. Picture credits: J.M.

**Figure 3:**
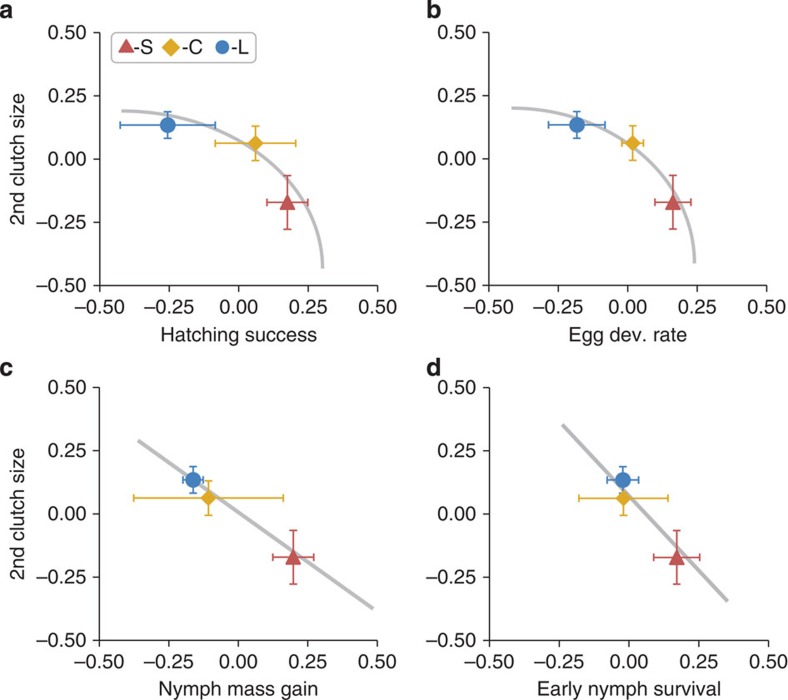
Shape of genetic trade-offs between second (2nd) clutch size and offspring performance. Shown are the trait means (±s.e.m.) from the last generation (generation six) across the S-lines (red symbols; *N*=4 lines, *n*=134 families), the C-lines (yellow symbols; *N*=2 lines, *n*=73 families) and the L-lines (blue symbols; *N*=4 lines, *n*=145 families). Curved trade-offs with diminishing returns before hatching for (**a**) hatching success and (**b**) egg developmental (dev.) rate. Linear trade-offs with constant returns after hatching for (**c**) nymph mass gain (slope (±s.e.)=−0.63 (0.01)) and (**d**) nymph survival (slope (±s.e.)=−1.37 (0.31)).

Despite genetic trade-offs, the evidence for conflict was weak after hatching when earwig mothers provide food to their young and nymphs signal their condition by solicitation pheromones^32^. The trade-off curves with nymph mass gain and survival were approximately linear indicating constant returns. Under constant returns, scope for conflict is limited and, if it is predicted, it is not over the partitioning of the amount of PI, but over whether or not the mother produces a second clutch (Fig. 1). The slope of the trade-off line was less steep than −1 for mass gain, which implies that with regard to effects on this offspring trait, earwig mothers and nymphs agree that females should not produce a second clutch (which could explain why a fraction of earwig females produces only one clutch in their lifetime^24^). For nymph survival the slope was steeper possibly in the range of mother–offspring conflict over second-clutch production. Indeed, our former research demonstrated that nymphs can influence whether or not caring females produce a second clutch, mediated by a paternally inherited effect^35^. Thus, whether or not earwig females produce a second clutch may have partly evolved due to the genetic trade-offs with nymph growth and survival. Our result that scope for conflict is more limited after than before hatching is somewhat surprising because parental feeding and offspring begging is the classical context used to model how parents and offspring should behave to resolve conflict^5^,^7^,^8^,^12^,^13^, where diminishing returns are commonly assumed, and thus the one where conflict over the amount of PI is *a priori* most expected.

By selecting on the relative size of second clutches, we focused on genetic trade-offs operating between clutches, which can drive conflict over PI among successive breeding attempts as originally envisaged by Trivers^2^. S-line nymphs evolved towards a higher mean offspring performance, without a significant change in the size of first clutches, which confirms the prominent role of the between-clutch trade-off for PI and scope for this form of conflict in *F. auricularia*.

Our findings highlight that the nature of conflict depends on genetic trade-offs and that conflict is not inevitable. Parent and offspring behaviours may also be driven by antagonistic mother–offspring co-evolution with no or minor influences of conflict. Such a process should result in co-adapted^17^,^36^ and well-coordinated parenting with low-cost honest begging^6^. Thereby, the genetic link between parental reproduction and offspring performance allows PI to quickly evolve and adapt in a changing environment.

From a life-history perspective, the constant returns and weak evidence for conflict after hatching, as compared with the egg stage, may at least partly reflect the partial independence of earwig nymphs from their mother's care during this stage^23^. Partial independence may limit conflict as compared with systems where offspring are fully dependent on their parents, such as altricial birds or mammals. Under partial independence, constant returns may be more likely because low levels of care have less devastating effects on offspring performance than under full offspring dependence and obligate care. If correct, this hypothesis would imply that parent–offspring conflict had limited impact in the early evolution of parenting when offspring did not fully depend on their parents and that, if present, conflict was mainly over whether or not parents should reproduce again (that is, their parity). More generally, the biological importance of genetic conflict should depend on factors determining the curvature of the genetic trade-offs shaping PI such as the life history and possibly also ecology of a population/species.

In conclusion, our study shows clear evidence for a genetic conflict between parents and offspring over PI. It thereby solves a long-standing problem that was previously conceived prohibitively difficult to address and, thus, fills a major gap in our empirical proof of concepts in the evolution of behaviours in families. Furthermore, and contrary to former thought, our results also reveal that conflict may not globally and *a priori* be assumed to be the major driver of parenting and family life. The nature and scope for conflict critically depends on the shape of the genetic trade-offs underlying PI, which needs empirical testing, and PI may also evolve by conflict-free antagonistic parent–offspring co-evolution enabling PI to evolve as coadapted and well-coordinated parenting and family life.

## Methods

### Laboratory breeding

The animals forming the base population of this selection experiment were caught from a wild population in early June 2009 in Dolcedo, Liguria/Italy (7° 56′ 55′′ E, 43° 54′ 14′′ N, altitude 78 m a.s.l.). It consisted of ∼1,200 predominately fourth juvenile instars and recently emerged adults. After transfer to the laboratory, the field-caught individuals were assigned randomly to 20 mating groups of 60 individuals each (30 females and 30 males) and kept separately in plastic containers for mating (see ref. 24 for a detailed description of the base population). The artificial selection experiment was initiated based on the progeny of these field-caught animals, that is, the first laboratory-born generation of adults (F1). Upon emergence as adults, the F1 males and females were randomly assigned to 20 mating groups of 48 individuals each (24 females and 24 males). The mating groups were held in plastic containers (dimensions: 37 × 22 × 25 cm) with humid sand as substrate and with egg cardboard and plastic tubes as shelters. The containers were lined with fluon and covered with nylon thighs to prevent escape of the animals. They were fed with our standard laboratory food (a food jelly made from 20 g egg yolk, 60 g wheat germ, 120 g carrots, 60 g bird food, 60 g dry cat food, 60 g flower pollen, 40 g Agar, 1,800 ml water, 2 g ascorbic acid and 2 g sorbic acid) with adequately sized pieces twice a week (see also ref. 24).

The mating groups were held in climate chambers at a light:dark photoperiod schedule of 14:10 h and at a constant temperature of 20 °C (to which we refer as ‘summer conditions') with relative humidity kept between 60 and 80%. As soon as at least two females from two different mating groups laid eggs, all females from all mating groups were set-up individually in Petri dishes (10 × 2 cm). The dishes contained humid sand as a substrate and a plastic tube as shelter. All females were kept for 7 days at 10 °C (no light) and then at 15 °C (no light) for oviposition and for the duration of egg care until hatching. Such ‘winter conditions' are required to terminate the diapause of the eggs and trigger embryonic development^23^,^24^. Each female was provided food twice a week until oviposition, and no food was provided during egg care until hatching^23^. On day 1 after hatching, we set-up the hatched nymphs with their mother in a new Petri dish (10 × 2 cm) and returned them to ‘summer conditions' (see above). During the first 2 weeks after hatching (that is, from day 1 until day 14), food was provided every other day. On day 14, females were separated from their nymphs and set-up in a new Petri dish (10 × 2 cm) for production of the second clutch (if any). Also on day 14, a total of 20 of her nymphs (or fewer in case of smaller nymph numbers) were chosen haphazardly and set-up in larger Petri dishes (14.5 × 2 cm) where they were reared as family groups until adulthood. After day 14, both females and nymphs were fed twice a week.

If a female produced no second clutch within 60 days after hatching of the first clutch, the female was considered to produce only one clutch in her lifetime^24^. If the female produced a second clutch, we took the performance measures of second-clutch offspring up until hatching (see section ‘Trait measurements' below). We did not rear any second-clutch offspring into adulthood. These basic procedures were applied to all generations of the selection experiment. The selection experiment was carried out over the course of six generations between spring 2010 and fall 2013.

### Experimental design

**Figure 4:**
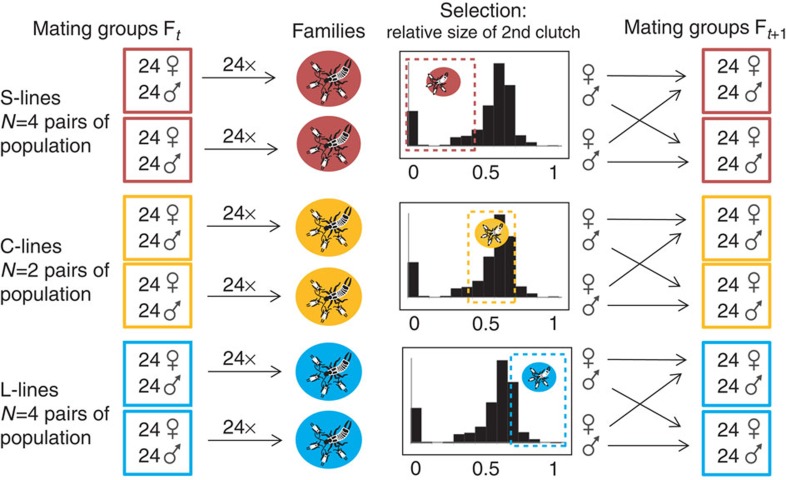
Illustration of breeding design. Each box to the left represents a mating group in generation F_*t*_, and to the right the mating group in the next generation F_*t*+1_. Two mating groups together (randomly assigned at begin of experiment) formed one population pair (that is, selection line) each, out of a total of ten population pairs. Red boxes, S-line; yellow boxes, C-line; blue boxes, L-lines. In generation *t* females, the distribution of the relative size of their second clutches was assessed for each mating group (histograms). Sons and daughters were then selected according to their mother's value for the selection target (selection depicted by dashed frames). The new mating groups in generation *t*+1 were formed of the daughters from the females from that same mating group in the previous generation, and the sons of the females from the other mating group of this population pair to prevent brother–sister mating. The experiment was run over six generations.

A graphical illustration of the experimental design can be found in Fig. 4. The selection experiment was initiated after one generation of laboratory breeding without selection to reduce a potential impact of environmental variation modifying the response to selection through maternal effects^37^. Of each brood produced by the 24 F1 females of each of the 20 mating groups, a female and a male were randomly selected to form the new 20 mating groups. The number of individuals per mating group was 24 females and 24 males across all generations of the selection experiment. To avoid brother–sister mating and minimize potential effects of inbreeding depression due to sib-mating, the 20 mating groups were randomly assigned into paired populations among which the females and males were exchanged each generation to form the mating groups of the next generation. For example, the female progeny of former population A were set-up with the male progeny of former population B to form the new population A (and *vice versa* for the new population B). The assignment of mating groups into population pairs was established at set-up of the field-caught individuals (F_0_) and was maintained over the whole course of the selection experiment. In this selection design, the unit of replication (that is, the selection line) is the paired population as it defines the independent gene pools that may evolve in response to selection.

From the total of 10 population pairs (that is, replicate selection lines), four were selected for a relatively small second clutch (‘S-lines'), four for a relatively large second clutch (‘L-lines') and two for an intermediate relative size of the second clutch (control ‘C-lines'). The relative size of the second clutch was computed as the number of eggs in the second clutch divided by the sum of eggs in the first and second clutches (the sum corresponding to the lifetime number of eggs in *F. auricularia*^24^). In the S-lines, we selected the bottom 50% (including females producing a single clutch), in the L-lines the top 50% and in the C-lines the intermediate 50% of the distribution in the relative size of second clutches among females of each mating group.

Although the relative size of the second clutch is a maternal trait with sex-limited expression, we applied selection through both sexes by using sons and daughters of the selected females/families (Fig. 4). We aimed at selecting two sons and two daughters of each selected female/family to keep mating groups of constant size (that is, 24 females and 24 males). This was not always possible due to cases of juvenile mortality, hatching failure or insufficient individuals from both sexes upon adult emergence in some of the families. In these cases, the number of selected individuals per brood/sex was adjusted by balancing stronger selection (using more individuals from mothers with the best fit to the selection criterion) against maintenance of genetic variability (using individuals from as many families as possible). The mean (±s.d.) numbers of females and males per family used over the six generations were 2.46 (0.86) and 2.50 (0.91), respectively. Only progeny from first clutches were used for breeding.

### Trait measurements

We took various measures of offspring performance including estimates of survival (separate for eggs/embryos and nymphs), estimates of developmental rate (separate for eggs/embryos, early nymphs (hatching—second instar) and late nymphs (second instar—adulthood)) and estimates of growth (separate for body mass at hatching and body mass gain during the first 14 days after hatching, as measure of growth after hatching). Survival is a direct component of fitness, and mass gain and fast development gives nymphs a headstart in competitive/cannibalistic interactions^38^,^39^. In addition, a range of reproductive parameters was recorded. The oviposition and hatching dates for first and second clutches were taken upon observation of the first eggs of a female and corresponded to the date of first observation of egg laying or hatching in a given clutch, respectively. Clutch sizes were determined by counting the number of eggs of the first and second clutches for each female 1 day after the first observation of the start of oviposition. Similarly, the number of hatched nymphs was counted 1 day after observation of the first hatched nymph in a clutch. Because hatching is sometimes asynchronous, the unhatched eggs were kept for another day to count further hatched nymphs (if any) on the subsequent day, and the number of unhatched eggs was also counted. The total number of hatched nymphs over the 2 days as proportion of clutch size was used to quantify hatching success.

Earwig females sometimes cannibalize some of their eggs during the period of egg care^34^. To obtain a quantity of egg cannibalism, the sum of the hatched nymphs and remaining unhatched eggs at hatching was compared with the original clutch size. Any reduction in the number of eggs between oviposition and hatching is most likely due to maternal egg cannibalism, and the difference in progeny number between oviposition and hatching was used as a measure of filial egg cannibalism in the analysis.

The body mass of nymphs was measured twice, 1 day after hatching and on day 14 after hatching. For each clutch, ten haphazardly chosen nymphs were jointly added to an Eppendorf tube and the tube was weighed with and without the nymphs. The difference divided by ten was taken as the average nymph body mass of a given clutch. Hatchling body mass was taken in all generations. Body mass at day 14 was only available for generations F1, F2, F3 and F6. The relative mass gain of nymphs over the course of the first 2 weeks after hatching was calculated as the proportional increase in mass relative to the body mass at hatching. We also took two measurement of female body mass, once at hatching and once 14 days after hatching. The weight gain of females from hatching of the first clutch until day 14 is a predictor for the likelihood and size of the second clutch^24^. All mass measurement were done to the nearest 0.01 mg using a Mettler-Toledo MT5 Micro-balance (Mettler, Roche, Basel). For measures of developmental rate we calculated the number of days between egg laying and hatching (egg developmental rate), the number of days between hatching and the first nymph in a clutch molting into second instar (early nymph development), and the number of days between second instar to the first adult emergence in a clutch (late nymph development).

### Statistical analysis

All variables were standardized to a mean of zero and unit variance within each generation for homogeneous variances across generations. To test for divergence of maternal and offspring traits between selection lines, we estimated standardized linear response gradients over the course of the six generations using linear mixed models and restricted maximum likelihood estimation. The trait of interest (standardized) was entered as the dependent variable, the selection treatment as fixed factor (H-lines, C-lines and L-line), the generation as continuous variable (linear term), the interaction between the selection treatment and generation as fixed factor and the paired populations (for example, ‘A–B') as random effect. A linear response to selection is in this model demonstrated by a significant interaction between the selection treatment and generation. The regression coefficients from this interaction term are standardized linear response gradients, that is, the slopes of the linear trend for the S- and L-lines relative to the control C-line. Standardized response gradients estimate the per-generation change in population mean trait values expressed in units of s.d.. The random effect (the paired population) accounted for the dependencies of individuals from the same selection line (that is, sharing the same gene pool) and for differences between lines within selection treatments arising for reasons other than selection as, for example, genetic drift. Proportional variables (relative size of second clutches, hatching success and nymph survival) were logit-transformed^40^ before standardization and analysis, and measures of developmental rate were computed by multiplying the standardized values of duration (number of days) by minus one, such that large positive values corresponded to fast development and large negative values to slow development. All reported *P* values are two tailed with a significance threshold *α* of 0.05. The statistical analyses were carried out using JMP PRO V11.0 statistical software (SAS Institute, Inc.).

## Author contributions

M.K. conceived the study and analysed the data; M.K. and J.M. designed the experiment and wrote the manuscript; S.B., J.M., J.W.Y.W., L.R. and D.S. managed the selection lines; and all authors collected the data and contributed to manuscript revisions with comments.

## Additional information

**How to cite this article:** Kölliker, M. *et al.* Parent–offspring conflict and the genetic trade-offs shaping parental investment. *Nat. Commun.* 6:6850 doi: 10.1038/ncomms7850 (2015).

## Figures and Tables

**Table 1:** Direct response to selection in earwig mothers and correlated responses to selection in their offspring.

	**Selection treatment**				**Generation**				**Interaction**				**Pop-pair**	**Response gradients (±s.e.)**	
	***df1***	***df2***	***F***	***P***	***df1***	***df2***	***F***	***P***	***df1***	***df2***	***F***	***P***	**Varcomp (±s.e.)**	**S-lines**	**L-lines**
Female															
Relative size of second clutch	2	7.04	10.23	**0.008**	1	2,278	0.02	0.887	2	2,279	7.54	**0.001**	0.0025 (0.0036)	−0.051 (0.017)	0.055 (0.017)
First-clutch size	2	7.03	2.35	0.165	1	2,707	0.16	0.692	2	2,707	0.58	0.560	0.0190 (0.0121)	−0.003 (0.015)	−0.015 (0.015)
Second-clutch size	2	7.06	2.08	0.195	1	2,277	0.02	0.883	2	2,277	3.82	**0.022**	0.0049 (0.0037)	−0.028 (0.012)	0.023 (0.012)
Likelihood second clutch	2	7.08	0.11	0.900	1	2,706	0.03	0.867	2	2,707	0.42	0.657	0.0033 (0.0037)	−0.014 (0.016)	0.005 (0.016)
Lifetime egg number	2	7.07	0.40	0.684	1	2,277	0.15	0.697	2	2,277	0.64	0.530	0.0148 (0.0092)	−0.013 (0.013)	0.008 (0.013)
Egg cannibalism*	2	7.03	0.16	0.858	1	2,707	0.15	0.694	2	2,707	3.92	**0.020**	0.0058 (0.0051)	−0.019 (0.016)	0.043 (0.016)
Body mass (d1)	2	7.06	1.08	0.389	1	2,504	0.16	0.684	2	2,504	2.64	0.072	0.0080 (0.0063)	0.036 (0.016)	−0.015 (0.016)
Mass gain (d1–d14)	2	7.20	2.39	0.160	1	2,454	0.30	0.584	2	2,454	6.68	**0.001**	0.0035 (0.0040)	−0.056 (0.016)	0.029 (0.016)
															
Offspring															
Hatching success	2	7.07	0.25	0.783	1	2,616	0.00	0.976	2	2,617	6.24	**0.002**	0.0023 (0.0033)	0.044 (0.016)	−0.045 (0.016)
Egg developmental rate	2	7.05	1.44	0.300	1	2,507	0.03	0.861	2	2,507	7.40	**0.001**	0.0062 (0.0054)	0.042 (0.016)	−0.054 (0.016)
Early nymph survival	2	7.02	0.88	0.458	1	2,462	0.74	0.390	2	2,462	4.50	**0.011**	0.0017 (0.0031)	0.046 (0.016)	0.002 (0.016)
Early nymph developmental rate	2	7.04	4.02	0.068	1	2,426	0.20	0.655	2	2,426	2.07	0.127	0.0034 (0.0040)	−0.008 (0.016)	0.033 (0.016)
Late nymph developmental rate	2	6.89	1.21	0.354	1	2,215	0.07	0.795	2	2,215	2.29	0.102	0.0163 (0.0110)	0.017 (0.017)	−0.035 (0.017)
Hatchling body mass	2	6.99	0.37	0.707	1	2,494	0.00	0.972	2	2,494	5.72	**0.003**	0.0274 (0.0164)	−0.041 (0.016)	0.044 (0.016)
Nymph mass gain†	2	7.21	2.83	0.124	1	1,405	0.02	0.897	2	1,405	3.37	**0.035**	0.0035 (0.0055)	0.038 (0.018)	−0.036 (0.018)

Results from linear mixed models (LMMs) on the standardized variables with the selection treatment as fixed factor, generation as linear covariate and the population pair as random effect. Data from six generations, *N*=10 population pairs (that is, selection lines) and a total of *n*=2,720 females (that is, families). Denominator degrees of freedom (*df*2) of different models may vary due to missing values of corresponding measurements. Provided are significance tests for the fixed effects and variance component estimates (±s.e.) for the random effect. Standardized response gradients were obtained as the regression coefficients from the interaction term between selection treatment and generation. They represent the linear slopes for S- and L-lines relative to the C-lines in units of s.d.. Significant (*α*=0.05) *P* values are in bold.

^*^Female cannibalism of eggs was calculated as the difference in the number of eggs between oviposition and hatching^34^. Egg number at hatching was the sum of hatched nymphs and unhatched eggs.

^†^Data only available for F1, F2, F3 and F6 generations.
